# CD24+CD44+CD54+EpCAM+ gastric cancer stem cells predict tumor progression and metastasis: clinical and experimental evidence

**DOI:** 10.1186/s13287-023-03241-7

**Published:** 2023-02-03

**Authors:** Angel A. Gómez-Gallegos, Lizbeth Ramírez-Vidal, Jared Becerril-Rico, Elizabeth Pérez-Islas, Zuly J. Hernandez-Peralta, Mariel E. Toledo-Guzmán, Alejandro García-Carrancá, Elizabeth Langley, Angélica Hernández-Guerrero, Fernando López-Casillas, Roberto Herrera-Goepfert, Luis F. Oñate-Ocaña, Elizabeth Ortiz-Sánchez

**Affiliations:** 1 Posgrado en Ciencias Biológicas, Unidad de Posgrado, Edificio A, 1° Piso, Circuito de Posgrados, Ciudad Universitaria, C.P. 04510 Coyoacán, Distrito Federal, Mexico; 2 Subdirección de Investigación Básica, Instituto Nacional de Cancerología, Av. San Fernando 22, Colonia Seccion XVI, Tlalpan, 14080 Mexico City, Mexico; 3 Posgrado de Ciencias Biomédicas. Facultad de Medicina, Universidad Nacional Autónoma de México, Circuito Exterior s/n Ciudad Universitaria, Coyoacán, 04510 Mexico City, Mexico; 4 Departamento de Patología, Instituto Nacional de Cancerología, Av. San Fernando 22, Colonia Sección XVI, Tlalpan, 14080 Mexico City, Mexico; 5 Unidad de Investigación en Cáncer, Instituto de Investigaciones Biomédicas, Universidad Nacional Autónoma de México, 04510 Mexico City, Mexico; 6 Unidad de Endoscopia, Instituto Nacional de Cancerología, Av. San Fernando 22, Colonia Sección XVI, Tlalpan, 14080 Mexico City, Mexico; 7 Instituto de Fisiología Celular, Universidad Nacional Autónoma de México, Circuito Exterior s/n Ciudad Universitaria, Coyoacán, 04510 Mexico City, Mexico; 8 Subdirección de Investigación Clínica, Instituto Nacional de Cancerología, Av. San Fernando 22, Colonia Sección XVI, Tlalpan, 14080 Mexico City, Mexico

**Keywords:** Gastric cancer, Cancer stem cells, Immunophenotype, Metastasis, Zebrafish, Xenotransplants

## Abstract

**Background:**

Gastric cancer (GC) is a leading cause of cancer-related deaths worldwide. Specific and thorough identification of cancer cell subsets with higher tumorigenicity and chemoresistance, such as cancer stem cells (CSCs), could lead to the development of new and promising therapeutic targets. For better CSC identification, a complete or extended surface marker phenotype is needed to provide increased specificity for new cell targeting approaches. Our goal is to identify and characterize a putative extended phenotype for CSCs derived from patients with GC before treatment, as well as to evaluate its clinical value. In addition, we aim to ensure that cells with this phenotype have stemness and self-renewal capabilities.

**Methods:**

This is a cohort study including 127 treatment-naïve patients with GC who attended the Instituto Nacional de Cancerología. Multiparametric flow cytometry analysis was performed to determine the extended phenotype of cells derived from gastric biopsies. The tumorigenic capability of cells identified in patients was assessed in a zebrafish model.

**Results:**

CD24+CD44+CD54+EpCAM+ cells were present in all treatment-naïve patients included, with a median abundance of 1.16% (0.57–1.89%). The percentage of CD24+CD44+CD54+EpCAM+ cells was categorized as high or low using 1.19% as the cutoff for the CD24+CD44+CD54+EpCAM+ cell subset. Additionally, a higher TNM stage correlated with a higher percentage of CD24+CD44+CD54+EpCAM+ cells (Rho coefficient 0.369; *p* < 0.0001). We also demonstrated that a higher percentage of CD24+CD44+CD54+EpCAM+ cells was positively associated with metastasis. The metastatic potential of these cells was confirmed in a zebrafish model. Ultimately, under our conditions, we conclude that CD24+CD44+CD54+EpCAM+ cells are true gastric cancer stem cells (GCSCs).

**Conclusion:**

The CD24+CD44+CD54+EpCAM+ cells present in tissue samples from patients are true GCSCs. This extended phenotype results in better and more specific characterization of these highly tumorigenic cells. The relative quantification of CD24+CD44+CD54+EpCAM+ cells has potential clinical value, as these cells are associated with metastatic disease, making their presence an additional prognostic marker and possibly a target for the design of new antineoplastic treatments in the era of precision oncology. Overall, the extended CD24+CD44+CD54+EpCAM+ phenotype of GCSCs could support their isolation for the study of their stemness mechanisms, leading to the identification of better molecular targets for the development of both new therapeutic approaches such as oncoimmunotherapy and new diagnostic and clinical prognostic strategies for GC.

**Supplementary Information:**

The online version contains supplementary material available at 10.1186/s13287-023-03241-7.

## Background

Gastric cancer (GC) is one of the most frequent cancers, ranking fifth in incidence and third in mortality among all cancers worldwide [1, 2]. GC was responsible for more than a million new cancer cases and over 768,793 deaths in 2020 [3]. Resistance to antineoplastic treatments in cancer patients can occur via the additive or synergistic effects of several mechanisms, including the presence of chemo- and radioresistant cells with high tumorigenic capacity, which are called cancer stem cells (CSCs). Several studies have indicated that a treatment targeting CSCs could be a promising therapeutic strategy. CSCs are a subpopulation of tumor cells that can self-renew and have cell differentiation potential [4–6]. Despite the evident differences between normal stem cells and CSCs, some similarities can be exploited to study CSCs, such as the expression of cell surface markers that allow CSC identification [7–9].

In general, the cell markers assessed to identify and isolate CSCs in most cancers, including GC, have been evaluated in cell lines with a single surface marker or a combination of two surface markers [10]. In primary cultures from hematological and solid neoplasms, several different cell surface markers have been used to identify and isolate CSCs; nevertheless, it is still necessary to establish an extended oncogenic phenotype, with a greater number of markers, to clearly identify CSCs for each type of cancer [7, 11, 12]. In GC, cells with CD44+CD24+ and CD44+CD54+ phenotypes have been described by some authors [13]. Although CD44+CD24+ and CD44+CD54+ cells have clinical relevance in patients with GC, it is still necessary to determine whether these cells with self-renewal capability are true gastric cancer stem cells (GCSCs). Thus, additional approaches have been used to generate combinations of markers that could result in more accurate CSC identification for the design and improvement of targeted therapies such as oncoimmunotherapy, which could decrease side effects that impact the lifespan and quality of life of cancer patients.

In addition to evaluating the presence of CSCs with an extended phenotype in samples from patients with primary GC, which could enable more specific identification, we aimed to determine the relationship of these highly tumorigenic cancer cells with prognosis and metastasis in patients with GC.

## Materials and methods

### Protection statement

The data presented in this manuscript are protected by the patent application MX/2018/007195-MX/E/2018/043906 submitted by Instituto Mexicano de la Propiedad Industrial (IMPI).

### Patients

This is a cohort study of 150 adult patients with GC who were treated at the Instituto Nacional de Cancerología in Mexico City between March 2015 and September 2019. Biopsies were collected before any antineoplastic treatment. The diagnosis of gastric adenocarcinoma was carried out by histopathologic evaluation of fresh biopsies obtained by endoscopy. Only 127 patients were included in the analysis due to the accessibility of clinical data and the quality of the tissue samples. Clinicopathologic characteristics, including age, sex, clinical TNM stage, Borrmann gross classification, Lauren’s classification, and differentiation grade based on WHO classification, were recorded in detail. Written informed consent was obtained from all patients. The study protocol was reviewed and approved by the “Comité de Investigación” and the “Comité de Bioética en Investigación” (research and bioethics committees) of the institute (Registration numbers 015/011/OMI; CEI/934/15 and 015/011/IBI; CEI/934/15).

### Sample processing for tumor cell suspension

Gastric biopsies (not surgical samples) were cut into small pieces with sterile scalpel blades. To obtain a single-cell suspension, the resultant minced tumor pieces were treated with 1 mg/ml ultrapure collagenase type IV (Worthington Biochemicals) and incubated at 37 °C for 1 h. The digestion mixture was washed twice with sterile PBS and centrifuged at 1200×*g* for 10 min. The mixture was then filtered through a 40 µm nylon mesh and washed with PBS to collect single cells.

### Cell line culture

The AGS GC cell line (ATCC, Rockville, MD, USA) was cultured in Ham-F12 media supplemented with 10% FBS. Tumorspheres were obtained by culturing 9 × 10^3^ cells/mL cells under nonadherent conditions in serum-free culture medium containing 2% B27 (Gibco) supplement, 10 ng/mL human bFGF (Sigma, Saint Louis, MO), and 20 ng/mL EGF (Invitrogen), followed by seeding in ultralow attachment plates (Corning) for 72 h under conventional cell culture conditions. The primary tumorsphere cells were gently dissociated, and the cells were used to evaluate the expression of surface markers. Cell viability was assessed by the trypan blue exclusion method. For the formation of secondary and tertiary tumorspheres, primary tumorspheres from 72 h of culture were harvested, gently dissociated and stained for FACS. Single-cell suspensions were used for culturing cells under the same conditions described above.

### Flow cytometry and cell sorting

Single cells were used for direct staining. Gastric tumor cells were passed through a 70 µm mesh filter (BD), and blood cells were removed by incubation in a solution containing KHCO3, 0.1 mmol/L EDTA, and 170 mmol/L NH4Cl for 10 min. AGS GC cells were stained with 5 µl per million cells of the following antibodies: CD24 (FITC, cat. 311104, BioLegend), CD44 (PE, 338808, BioLegend), CD45 (APC, 304037, BioLegend), CD54 (Pacific blue, 353110, BioLegend), CD73 (Pe-Cy7, 344009, BioLegend), CD90 (Brilliant violet 421, 328126, BioLegend), EpCAM (CD326) (Pe-Cy7, 234222, BioLegend), and STRO-1 (Pe-Cy5, 340106, BioLegend). After 25 min of incubation at 4 °C in the dark, the cells were rinsed twice with 0.5% BSA in PBS. For GC tissue samples, CD45+  cells were discarded from the analysis. After incubation, cells were acquired on an Attune Nxt® cytometer (Thermo Fischer) at the Laboratorio Nacional de Citometría, Instituto de Investigaciones Biomédicas (IIB), Universidad Nacional Autónoma de México (UNAM) (LabNalCit, IIB, UNAM). The acquisition data were analyzed with Flow Jo software (TreeStar). For cell sorting assays, cells were stained and sorted by flow cytometry using the MoFlo® Astrios (Beckman Coulter). Postsort analysis was performed to ensure that the purity of the cell fractions was > 95%. Cells were recovered in serum-free culture medium, washed twice with sterile PBS, and counted before reseeding.

### Zebrafish husbandry and lines

Adult zebrafish (*Danio rerio*) were obtained and maintained at 28.5 °C in the aquarium facility at Instituto de Fisiología Celular, UNAM (IFC, UNAM) according to standard procedures (Westerfield, 2007). The zebrafish model was kindly donated by Dr Javier Torres Vazquez from the Department of Cell Biology, NYU Grossman School of Medicine, USA. Zebrafish embryos were obtained from natural crosses, and we placed 1 male and 1 female adult zebrafish (6 to 18 months old) in individual rearing tanks in the Dr López-Casillas aquarium facility at the Instituto de Fisiología Celular, UNAM. All experiments were approved by the Committee for Laboratory Animal Care and Use of the IFC, UNAM, under CICUAL-Protocol number FLC139-18.

Transgenic fluorescent reporter embryos of the zebrafish line *Tg(fli1:EGFP)*^*y1*^ were staged on the basis of hours post-fertilization (hpf) according to Kimmel et al., 1995. Transgenic zebrafish embryos Tg(*fli*1:*EGFP*)^*y1*^, expressing EGFP in endothelial cells, were treated with phenylthiourea (PTU; 0.003% w/V; Sigma) to prevent pigmentation of the larvae. All animals were anesthetized with 164 mg/L tricaine (MS-222, Sigma) prior to euthanasia by chilling on ice.

### Microinjection of GCSCs into zebrafish embryos

Tumorsphere cells derived from the AGS cell line were first sorted using a fluorescence flow cytometer (MoFlo, Beckman Coulter), and subpopulations of fluorescence-emitting positive and negative cells were collected in a sterile tube. Then, the cells were stained with 1 µg/mL CM-DiI dye (Invitrogen, Life Technologies) according to the manufacturer’s instructions, washed three times in sterile PBS, and resuspended in fresh PBS. The cell density of the suspension was calculated based on cell counting with a hemocytometer and adjusted to 50 × 10^6^ cells/ml in PBS. Cell viability was assessed by trypan blue staining before injection.

On the day of injection, 48 hpf Tg(*fli*-1:*EGFP*)^*y1*^ zebrafish embryos were dechorionated and randomly separated into three groups of embryos. Embryos to be injected with GCEPs, GCnEPs, or PBS were anesthetized (164 mg/L tricaine) and injected in this order for all experiments. After cell injection, the embryos were incubated separately in appropriately labeled 100 mm Petri dishes with fresh water at 31 °C. Photographs or measurements were recorded from 15:00 to 19:00 h every day from the day after injection, starting with embryos injected with GCEPs.

The sample size was 300 embryos for each round of injection for a total of 4 rounds. The embryos were randomly divided into three groups (embryos injected with GCEPs, GCnEPs, and PBS as a control). For injection, all embryos were positioned with their right side up on an agarose pad with aquarium water.

In a simple comparative study for the evaluation of the tumorigenic capacity of GCEPs vs GCnEPs, 50 or 200 cells were injected into the yolk sac of each embryo using a microinjector (Femtojet express, Eppendorf) and a stereoscopic microscope (SMZ 745T, Nikon). After injection, the embryos were incubated for 4 h at 31 °C, and all embryos with fluorescent cells in the circulatory system were discarded. Therefore, the number of zebrafish embryos evaluated for each experiment was established after cell injection (± 40% of zebrafish embryos discarded).

Then, the embryos were incubated at 31 °C for the following days. As the temperature for embryo development is 27 °C and that for human cells is 37 °C, the embryos were incubated at 31 °C, a temperature that maintains embryo viability and allows human cells to proliferate in xenograft assays. Embryos injected with the same volume of PBS or that did not receive an injection were defined as experimental controls. The first coauthors (Ángel Arturo Goméz-Gallegos and Lizbeth Ramírez-Vidal) carried out each experiment, supervised the injection of the cells (GCEPs and GCnEPs as well as PBS) into the corresponding group of embryos, evaluated the results, and analyzed the data.

### Histological processing

Anesthetized larvae were fixed with 4% paraformaldehyde in PBS overnight at 4 °C and embedded in 15% sucrose-7.5% gelatin in PBS for cryosectioning (Leyca). Six, 10 or 15 µm transverse sections were obtained and mounted for direct observation of fluorescent signals or processed for hematoxylin and eosin (H&E) or periodic acid-Schiff–Alcian blue staining (PAS-AB). Staining was performed in the histology facilities of IFC, UNAM. The tissue sections for fluorescence analyses were stained with Hoechst dye to observe the nuclei.

### Imaging

We monitored in vivo tumor cell growth and migration by fluorescence for 6 days post-injection (dpi) on a Nikon SMZ150 stereomicroscope. The images of whole zebrafish larvae were acquired daily with a DS-Fi1 camera (Nikon) and version 4.3 of NIS Elements F software (Nikon) from 1 to 6 days post-injection (dpi). First, the image background was subtracted from each channel, and then, overlay was performed with FIJI (ImageJ) software. For whole-embryo images, we increased the signal intensity of stained GCSCs to make them visible at 2X magnification. Fluorescence images of whole larvae or cryosections were acquired with an LSM 800 Confocal Microscope (Carl Zeiss, batch number 2633000222) with GaAsP detectors and a Plan-Apochromat 20X/0.8 M27, Plan-Apochromat 40X/1.3 oil DIC(UV) VIS-IR M27 or Plan-Apochromat 63X/1.4 oil DIC M27 objective. Image acquisition and processing were performed using Carl Zeiss Zen Blue 2.3 software. We acquired a tiled array with x̄ = 1 and 2980 × 4914 pixels per image with a 20X objective, and then, we extracted single-slice images for the figures presented in this report. Nonprecessing was applied in all images included in this report, and only enhancement of the signal at the same level for each channel was applied to be able to visualize the images easily. Images acquired with a 63X objective were acquired as single images or a tiled array with ***x̄* = *1*** and 1437 × 1437 pixels per image. No processing was applied, and we only enhanced the signal at the same level for each channel.

Image acquisition of H&E- or AB-Pas-stained slides was carried out on a stereoscopic microscope AxioZoom V16 with an ApoTome (Carl Zeiss, batch number: 4633001353) and a PlanNeoFluar Z 2.3X/0.57 objective. Image acquisition was performed with Axiocam503 and Zen PRO software (Carl Zeiss). Figures were exported to Photoshop Cs6 (Adobe) for final editing and presentation.

### Statistical analysis

Data were summarized as frequencies and percentages. The cutoff for high and low % GCEPs was established at 1.19% based on the second quartile of the data set. For categorical variables, differences between groups were evaluated using the X^2^ test or Fisher’s test, as appropriate. Spearman’s correlation coefficient was evaluated as a measure of correlation between ordinal variables. The Kaplan‒Meier method was used for survival analysis, and the log-rank test was used to compare survival curves. Statistical analyses were performed employing IBM SPSS Statistics version 28 (IBM Corp., Armonk NY, USA, 2021), with two-tailed statistics and a critical value of *p* < 0.05.

### In vitro and in vivo assays

All statistical results are expressed as the mean and standard error of the mean (SEM) using GraphPad Prism 5.0. Decreases/increases in fold change were analyzed using one-way ANOVA. All experiments were repeated at least three times.

## Results

### Identification of cells with an extended stem cell surface phenotype in patients with GC

**Fig. 1:**
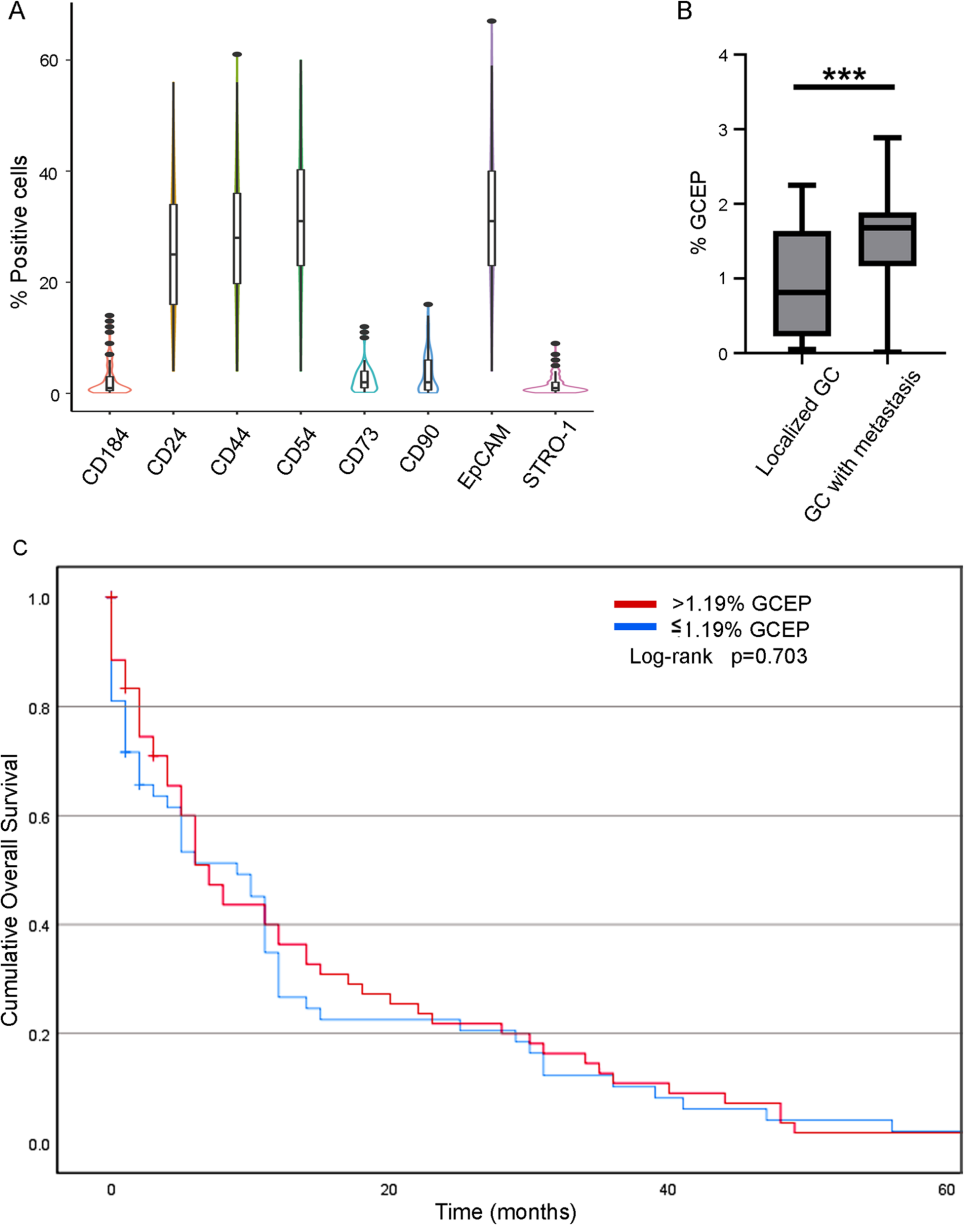
Determination of stem cell markers and their Relationship with the Clinical Outcome of Gastric Cancer Patients. **A** Evaluation of 8 surface markers related to CSC in CD45− cells from 127 biopsies of gastric cancer patients. Error bars represent ± SD. **B** Plots of CD24+CD44+CD54+EpCAM+ (GCEP) cell percentage in patients with gastric cancer with metastasis and patients with localized gastric cancer. Error bars represent ± SD. ***p < 0.001. **C** Kaplan–Meier survival graph of patients with high (> 1.19) or low (< 1.19) %GCEP. No significant differences were observed between groups in the Log-rank test

Only limited and variable CSC phenotypes have been reported in GC cells, mainly in cell lines [7, 10, 13, 14]. To more specifically identify and study GCSCs, we evaluated an extended panel of CSC markers in 127 gastric biopsies collected from patients with a diagnosis of GC who attended the Instituto Nacional de Cancerología in Mexico City from 2015 to 2019. First, we selected cells negative for leukocyte antigen (CD45) to discard hematopoietic cells [15]. Then, we evaluated eight different putative GCSC markers in each patient sample (Fig. 1A). We included the expression of the CD24 and CD44 markers since these are the most widely used markers to identify CSCs in GC cell lines [11]. Additionally, we measured the expression of the CD54, EpCAM, STRO-1, CD73, CD90, and CD184 markers, which have previously been suggested as single markers in GC cell lines [10–14].

We found that the expression of CD54 and EpCAM was significantly higher than that of STRO-1, CD73, CD90, and CD184 under our conditions (Fig. 1A). These results indicate that there is a subset of tumor cells in patients with GC that present a CD24+CD44+CD54+EpCAM+ surface marker combination, which we refer to as gastric cancer cells with an extended phenotype (GCEPs). This 4-marker combination was used for all subsequent experiments.

Next, we determined whether this same GCEP phenotype could be found in cells derived from 3D AGS cell line cultures. Using a CSC-enriched 3D cell culture of AGS GC cells, we measured GCEP markers in a time-dependent assay from day 0 to 7 (Additional file 1: Figure S1). On day three of culture, we found a higher proportion (± 20%) of GCEPs present in tumorspheres. Furthermore, as observed in the GC cells derived from patients, there was no codetection of the CD73, CD90, CD184, or STRO-1 markers in the CD24+CD44+CD54+EpCAM+ subpopulation in 3D AGS cell cultures (Additional file 2: Figure S2). Additionally, the GCEP phenotype was also found in the KATO and NCI-N87 GC cell lines (Additional file 3: Figure S3). These results confirm the presence of the same extended phenotype present in GC biopsies and tumorspheres derived from various GC cell lines.

Since the cell phenotype does not necessarily correlate with stemness, it was important to confirm that GCEPs are GCSCs. Due to the small number of cells obtained from tissue biopsies, AGS cells were used for subsequent experiments to determine stemness capabilities. To confirm the presence of stemness markers in GCEPs, the NANOG [16, 17], OCT4 [18, 19], and SOX2 [20–22] transcription factors (TFs) were evaluated in GCEPs derived from 3D AGS cell cultures (Additional file 4: Figure S4). We observed that more than 80% of GCEPs from AGS tumorspheres were positive for NANOG, OCT4, and SOX2.

**Fig. 2:**
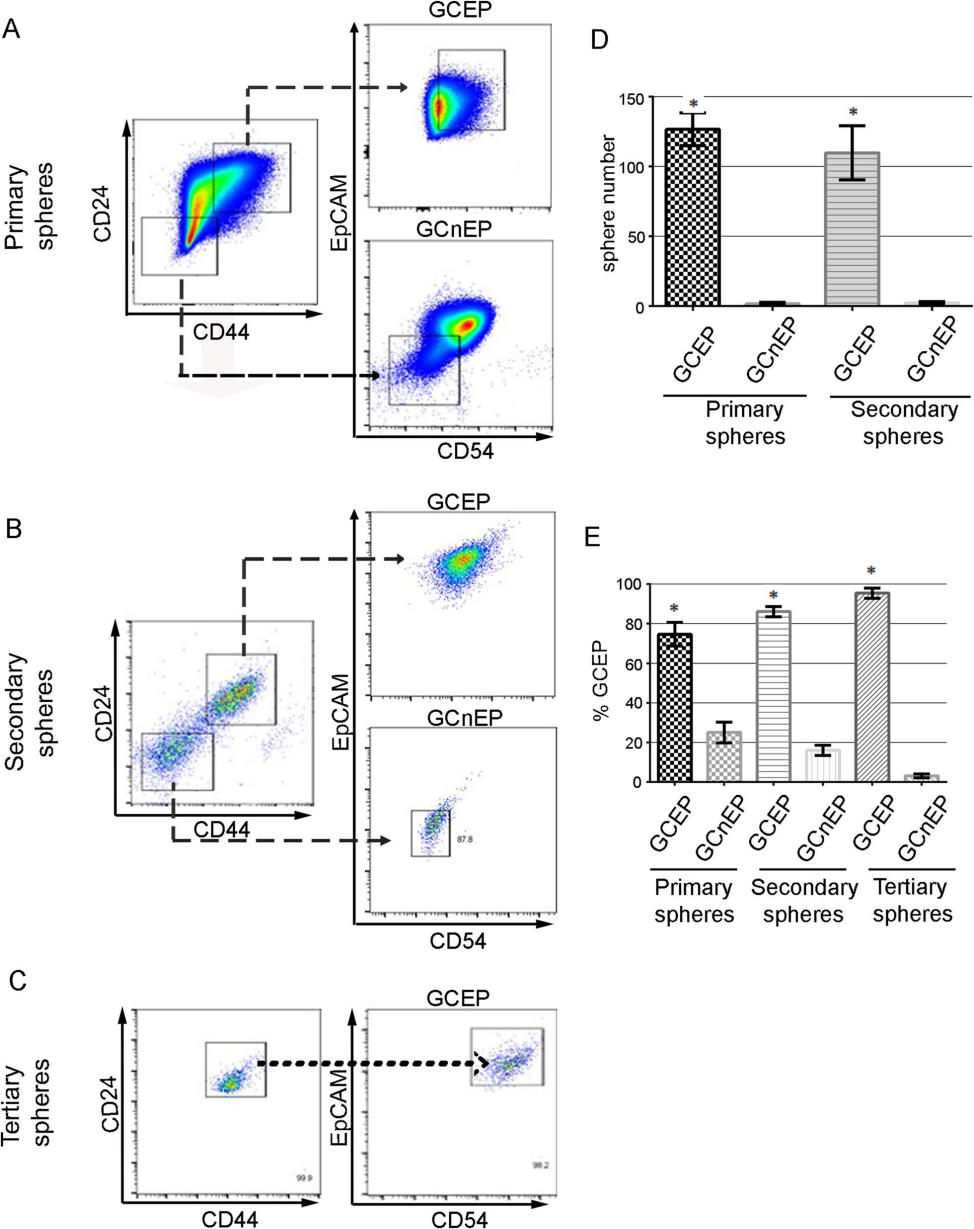
CD24+CD44+CD54+EpCAM+ cells show Cancer Stem cell properties. (**A**–**C**) Cell differentiation potential of GCEP (CD24+CD44+CD54+EpCAM+). Cells were able to generate different phenotypes under the same cell culture conditions. **A** Primary tumorspheres, **B** Secondary tumorspheres came from GCEP cells derived from primary tumorspheres. **C** Tertiary tumorspheres came from GCEP cells derived from secondary tumorspheres. **D** GCEP cells showed greater capacity to form secondary and tertiary tumorspheres after sorting. Only the GCEP cell subpopulation was able to form secondary and tertiary spheres. **E** Percentage of cells with GCEP and GCnEP (CD24−CD44−CD54−EpCAM−) cells present in primary, secondary, and tertiary tumorspheres. Error bars indicate the ± SD of three independent assays. *P ≤ 0.05

Self-renewal capacity is the most representative characteristic of stem cells [23]. To evaluate this capacity, GCEPs and cells negative for the extended phenotype (CD44−CD24−CD54−EpCAM− cells, GCnEPs) derived from primary AGS tumorspheres [24–26] (Fig. 2A) were challenged to form secondary (Fig. 2B) and tertiary tumorspheres (Fig. 2C) under nonadherent conditions. Unlike GCnEPs, GCEPs were able to form secondary and tertiary tumorspheres (Fig. 2D). Furthermore, these tumorspheres exhibited heterogeneous phenotypes, such as CD44+CD24+ cells, CD44−CD24− cells, and CD44+CD24− cells, indicating the presence of different subsets (Fig. 2B). The percentage of GCEPs compared with GCnEPs increased from primary to tertiary tumorspheres (Fig. 2E). In addition to expressing stemness transcription factors, these data indicate that GCEPs possess CSC characteristics, such as self-renewal and differentiation capabilities.

### The CD24+CD44+CD54+EpCAM+ cell phenotype is associated with clinical stage and metastasis in GC patients

In our patient cohort, 54.3% of patients were men, and 45.6% were women, with a median age of 56.1 years (44–65 age range). Patient clinical characteristics are shown in Table 1, and most patients had advanced stage GC. The mean follow-up period was 11.9 months (0–63 months). GCEPs were present in all GC patients, with a median level of 1.16% (0.57–1.89%). For relational analysis, the GCEP percentage (%GCEP) was categorized using 1.19% as the cutoff for high or low %GCEP. Differences in %GCEP were evident among groups divided by TNM clinical stage (Table 1). Additionally, the Spearman test also showed that a higher cancer stage correlated with a higher %GCEP (*p* < 0.0001, Rho coefficient 0.369). TNM analysis showed that patients with higher %GCEP were more likely to experience metastasis (Fig. 1B and Table 1), indicating the relevance of GCEPs for GC, specifically for the clinical stage and the degree of disease progression. Therefore, we hypothesized that CD24+CD44+CD54+EpCAM+ cells would have migratory functions during GC progression.

**Table 1:** Clinical characteristics of patients

Characteristics	*n* = 127 (%)	%GCEP	*p* value
Sex			0.585^a^
Male	69 (54.3%)	1.16% (0.36–1.83)	
Female	58 (45.6%)	1.13% (0.47–1.72)	
Age group			0.69^a^
Young adult	8 (6.3%)	1.28% (0.61–1.89)	
Middle-aged adult	67 (52.8%)	1.08% (0.32–1.78)	
Older adult	52 (40.9%)	1.19% (0.49–1.74)	
AJCC stage			< 0.0001^b^
0	1(8.0%)	0.55%	
I	4 (3.0%)	1.38% (0.54–1.92)	
IIA	16 (12.0%)	0.60% (0.14–0.93)	
IIB	8 (6.0%)	1.10% (0.24–1.98)	
III	14 (10.5%)	0.82% (0.17–1.70)	
Iva	34 (25.6%)	0.99% (0.35–1.69)	
IVb	50 (37.6%)	1.52% (1.22–1.88)	
cTNM stage			
T			0.247^b^
Tis	1 (0.8%)	0.55%	
T1	13 (10.2%)	1.01% (0.48–1.65)	
T2	11 (8.7%)	0.85% (0.19–1.60)	
T3	25 (19.7%)	1.53% (1.10–1.96)	
T4a	39 (30.7%)	1.07% (0.33–1.72)	
T4b	38 (29.9%)	1.15% (0.16–1.85)	
N			0.616^a^
N0	14 (11%)	1.35% (0.74–2.01)	
N1	34 (26.8%)	1.01% (0.30–1.77)	
N2	44 (34.6%)	1.18% (0.51–1.73)	
N3	35 (27.6%)	1.21% (0.39–1.82)	
M			< 0.0001^a^
M0	75 (59.1%)	0.91% (0.24–1.62)	
M1	52 (40.9%)	1.52% (1.17–1.88)	
Lauren’s classification			0.187^b^
Nonclassifiable	7 (5.5%)	0.78% (0.90–0.93)	
Intestinal	49 (38.6%)	1.13% (0.34–1.78)	
Diffuse	68 (53.5%)	1.21% (0.52–1.78)	
Mixed	3 (2.4%)	1.22% (0.85–1.80)	
Histological grade			0.259^a^
Nonclassifiable	6 (4.7%)	0.62% (0.90–1.43)	
Poorly differentiated	98 (77.2%)	1.00% (0.51–2.21)	
Moderately differentiated	17 (13.4%)	0.81% (0.34–1.93)	
Well differentiated	6 (4.7%)	0.72% (0.34–1.74)	
Borrmann’s classification			0.516^a^
Type 1	8 (6.3%)	1.43% (0.65–1.98)	
Type 2	8 (6.3%)	1.35% (0.49–1.98)	
Type 3	55 (43.3%)	1.23% (0.53–1.79)	
Type 4	41 (32.3%)	1.06% (0.26–1.73)	
Type 5	15 (11.8%)	0.88% (0.45–1.71)	

AJCC, American Joint Committee on Cancer

^a^Association between categorical %GCEP and clinical variables using *X*^2^ test

^b^Association using Fisher test

Age group classification was considered as: adolescence, 1–17 years old; young adult, 18–29 years old; middle-aged adult, 30–59 years old; and older adult, ≥ 60 years old

Despite these results, survival analysis did not show a difference between patients with high and low %GCEP (Fig. 1C), implying that overall survival is not dependent on %GCEP. Additionally, the Kaplan‒Meier graph shows higher mortality in the first year of follow-up, with more than half of the patients dying during this period, revealing the short-term lethality of GC in the patients evaluated.

We also analyzed the abundance of CD24+CD44+CD54+EpCAM+ cells in 45 samples from patients without GC. Our preliminary data show the presence of a subset of cells with a different cell surface phenotype (data not shown) in patients without GC compared to the GCEPs present in biopsies of patients with GC. However, it is necessary to evaluate more samples from patients without GC to obtain conclusive data about the diagnostic value of these GCEP markers. Currently, we are collecting data from a phase I diagnostic test study in our laboratory to corroborate these data.

### GCEPs possess high invasion and migration capabilities in vivo

To address tumorigenic capability, GCEPs and GCnEPs derived from 3D AGS cell cultures were injected into zebrafish embryos. After tumorsphere cell sorting, cells were labeled with a fluorescent cell tracker (CM-Dil) prior to in vivo xenotransplantation. Forty-eight hours post-fertilization (hpf), zebrafish embryos were injected with 200 GCEPs, GCnEPs, or PBS (negative control). At 4 h post-injection (hpi), embryos with few cells, mechanical damage, or cells outside the yolk sac were discarded. Embryos injected with 200 GCEPs showed hematogenous spread (Additional file 5: Figure S5A) in the head, trunk, and tail 1 day post-injection (dpi). Their survival rate was 17%, compared to 87% in embryos injected with GCnEPs and 95% in the PBS controls (Additional file 5: Figure S5B). Due to the toxicity observed using 200 GCEPs, we decided to carry out further experiments by injecting only 50 labeled GCEPs or GCnEPs into the yolk sacs of zebrafish embryos.

**Fig. 3:**
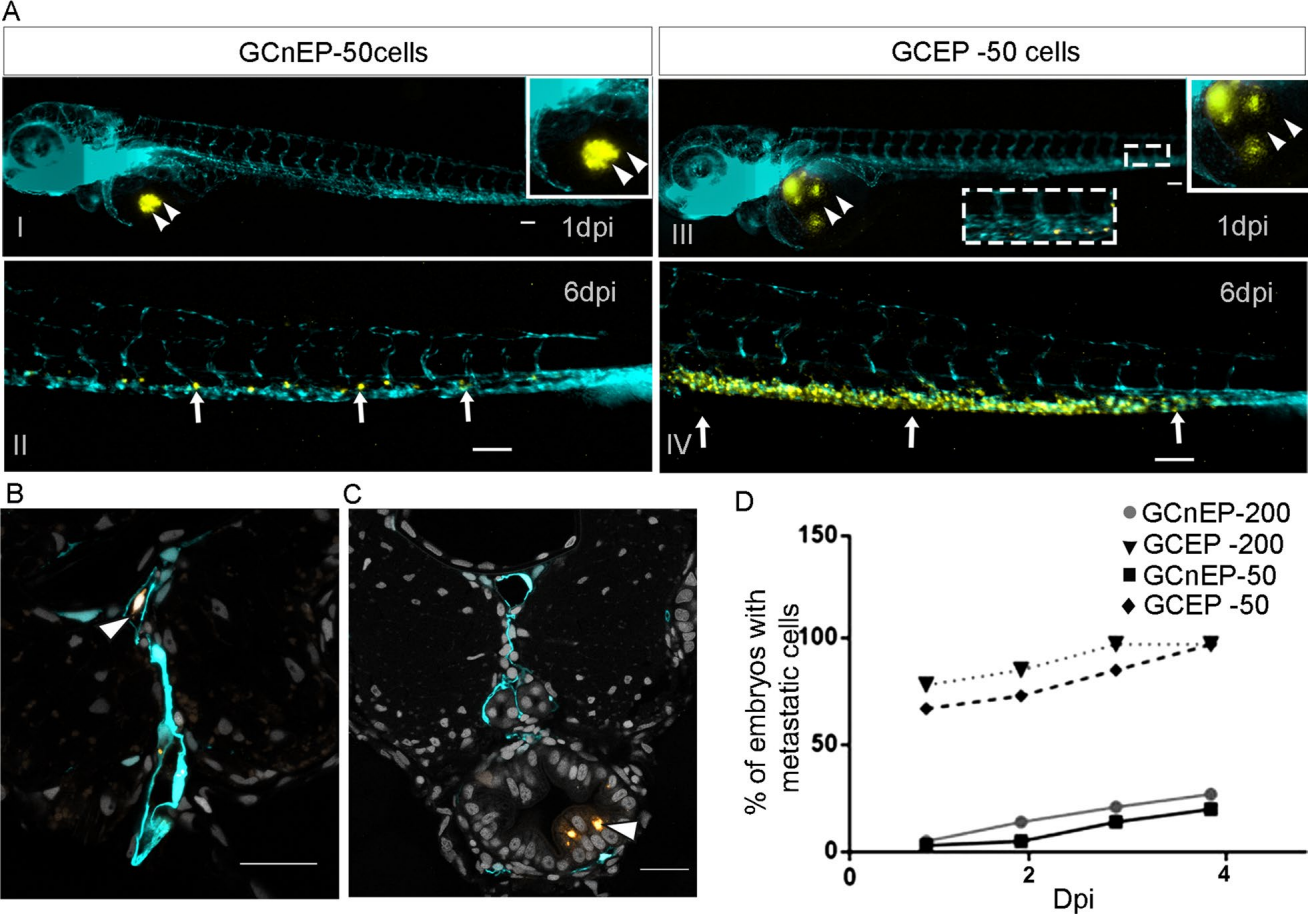
Xenotransplanted gastric cancer GCEP cells migrate and form metastasis in zebrafish. **A** Cell migration after 1 and 6 dpi of 50 GCnEP cells (panels I and II, respectively) or GCEP cells (panels III and IV, respectively). Double arrowheads and insets indicate the site of injection, arrows highlight the sites with cell migration. Cyan fluorescence marks vascular endothelium in Tg(*fli*-1:*EGFP*)^*y1*^ zebrafish transgenic larvae. **B**, **C** Transversal section showing the caudal portion of a 6 dpi Tg(*fli*-1:*EGFP*)^*y1*^ zebrafish larvae injected with GCEP cells. The white arrowhead shows a GCEP cell traveling inside the caudal artery in panel B. Arrowhead showing a metastatic tumor in the distal portion of the intestine at the level of the somites 14–16 in panel **C**. **D** Percentage of embryos with migration of cells at 1, 2, 3 and 4 days post-injection (dpi); embryos showing disseminated cells far from the sites of injection were counted. All image acquisition was carried out in a Nikon SMZ1500 stereomicroscope. All scale bars indicate 100 μM

We observed that GCEPs migrated from the yolk sac to the tail after 1 dpi [27] (Fig. 3A), exhibiting higher invasive capability than GCnEPs, which remained in the yolk sac (Fig. 3A I–II). From 1 to 6 dpi, more GCEPs continued to migrate from the yolk to the tail of the larvae (Fig. 3A III-IV). In contrast, GCnEPs were observed in the tail only after 6 dpi (Fig. 3A II), which shows that the latter have a weaker potential to migrate, in accordance with previous reports [28]. These results indicate that GCEPs display higher invasive capacity than GCnEPs. Notably, the GCEPs did not remain together but were scattered throughout the yolk sac, close to the subintestinal plexus of the embryo (double arrowheads in Fig. 3A panel III). Under confocal microscopy, tissue slices allowed us to observe GCEPs traveling inside a blood vessel (white arrowhead in Fig. 3B); some of these cells were able to extravasate the blood vessel and invade the distal portion of the intestine, forming a metastatic tumor at the level of somites 14–16 (white arrowhead in Fig. 3C). This demonstrates the ability of GCEPs to migrate in the circulatory system and extravasate from blood vessels to colonize distant sites [27, 29].

Furthermore, at 2 and 3 dpi, the percentage of larvae with GCEP migration was much higher than that of GCnEP-injected zebrafish (Fig. 3D).

### Invasion, migration, and tumor formation in GCEP-injected larvae

**Fig. 4:**
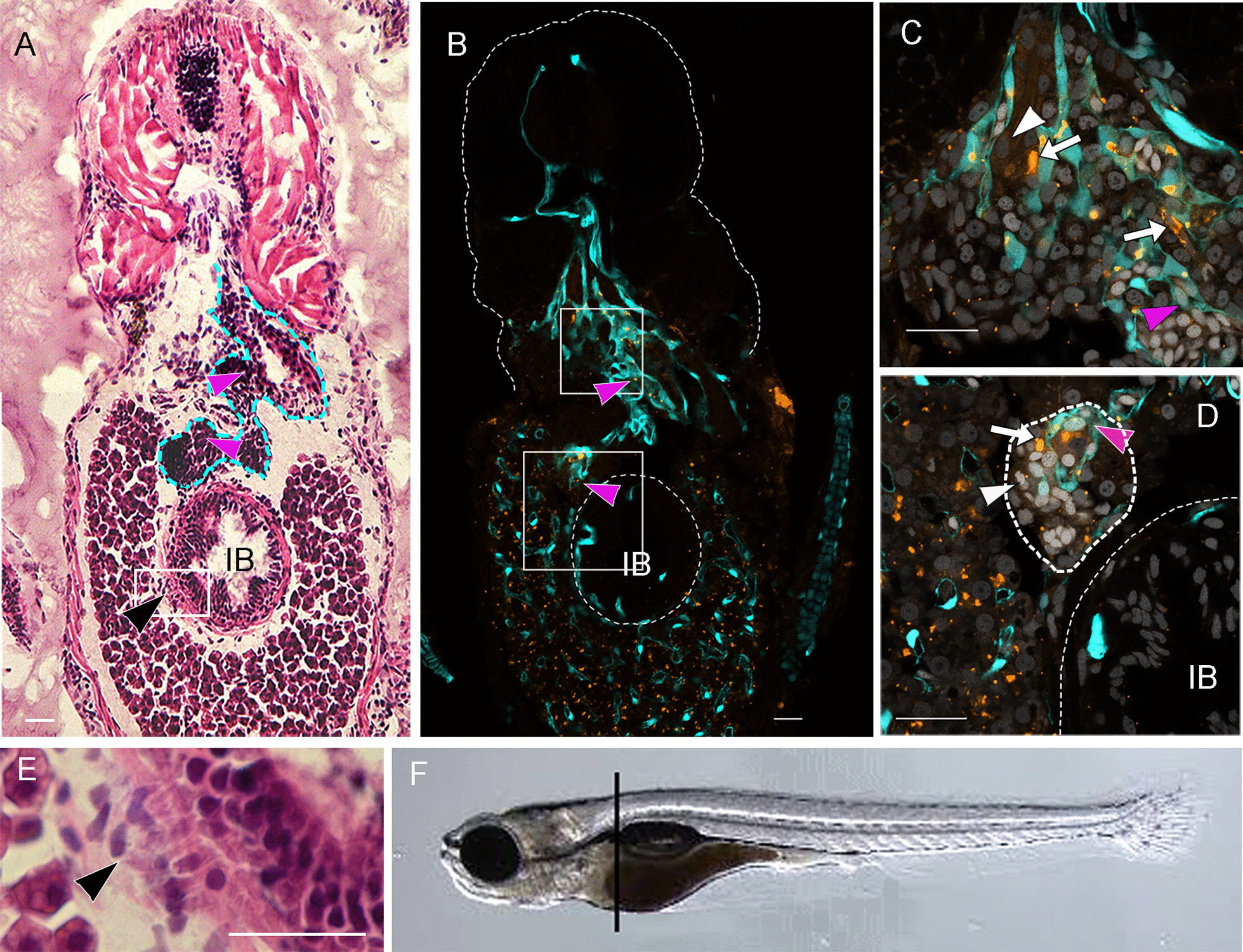
GCEP cells have invasion, migration, and proangiogenic capabilities. Transversal 15 mm sections at the trunk level of * fli-1* zebrafish larvae after 6 dpi with 50 GCEP cells. **A**, **E** H&E-stained sections; dashed line in 4A shows an aberrant vessel or a group of vessels, and black arrowhead shows cells moving from the yolk to the intestinal bulb (IB), inset in **E**. **B**–**D** Confocal images of transversal sections showing CM-Dil-stained GCEP cells in orange, cyan marking endothelial cells from a Tg(*fli*-1:*EGFP*)^*y1*^ zebrafish larvae, and Hoechst stained nuclei in light gray. Pink arrowheads indicate the large vessel observed in panel **A**, **B**. White arrows and arrowheads show injected cells with high and low fluorescence intensity, respectively. **C**, **D** insets indicated in panel B, dashed line in panel D showing an irrigated tumor. **F** side view of a larvae indicating sectioning site. All scale bars indicate 100 μM. Images were acquired with a Zeiss confocal microscope LSM800 or Axio Zoom V16, stereomicroscope

To determine whether the injected GCEPs and GCnEPs can induce tumor growth, angiogenesis and metastasis, the injected embryos were fixed and cryosectioned to observe fluorescently labeled cells or for staining with H&E to observe the tissue structure in more detail (Fig. 4).

Tissue sections at the level of the yolk (Fig. 4F) from transgenic Tg(*fli*1:*EGFP*)^*y1*^ larvae injected with 50 cells showed many GCEPs in the yolk (yellow/orange in the image) at the level of the intestinal bulb (IB) and near the injection site after 6 dpi (Fig. 4B–D). Pink arrowheads (Fig. 4A–D) and the cyan-dashed line in Fig. 4A show the formation of either a large, misplaced blood vessel or a group of vessels (the thickness of the slide did not allow us to clearly define which) growing toward a cell mass (dashed line and white arrowhead in Fig. 4D) near the IB. Neovascularization and a growing tumor can be observed in Fig. 4C, with cell nuclei in light gray (Hoechst staining). Figure 4A shows the cell structure of a contiguous section stained with H&E. Interestingly, some GCEPs were stained more intensely (white arrow in Fig. 4D) than others (white arrowhead in Fig. 4D), suggesting the presence of proliferating cells (Fig. 4C, D).

We also observed tumor formation and infiltration of GCEPs from the yolk to the IB (black arrowheads in Fig. 4A, E). The amplified area in Fig. 4A shows the changes in IB morphology and the infiltration of mesenchymal-like cells (black arrowhead). This demonstrates the capability of injected GCEPs to migrate long distances in the circulatory system and extravasate from blood vessels to colonize distant sites.

### GCEP and GCnEP phenotypes in injected zebrafish larvae

**Fig. 5:**
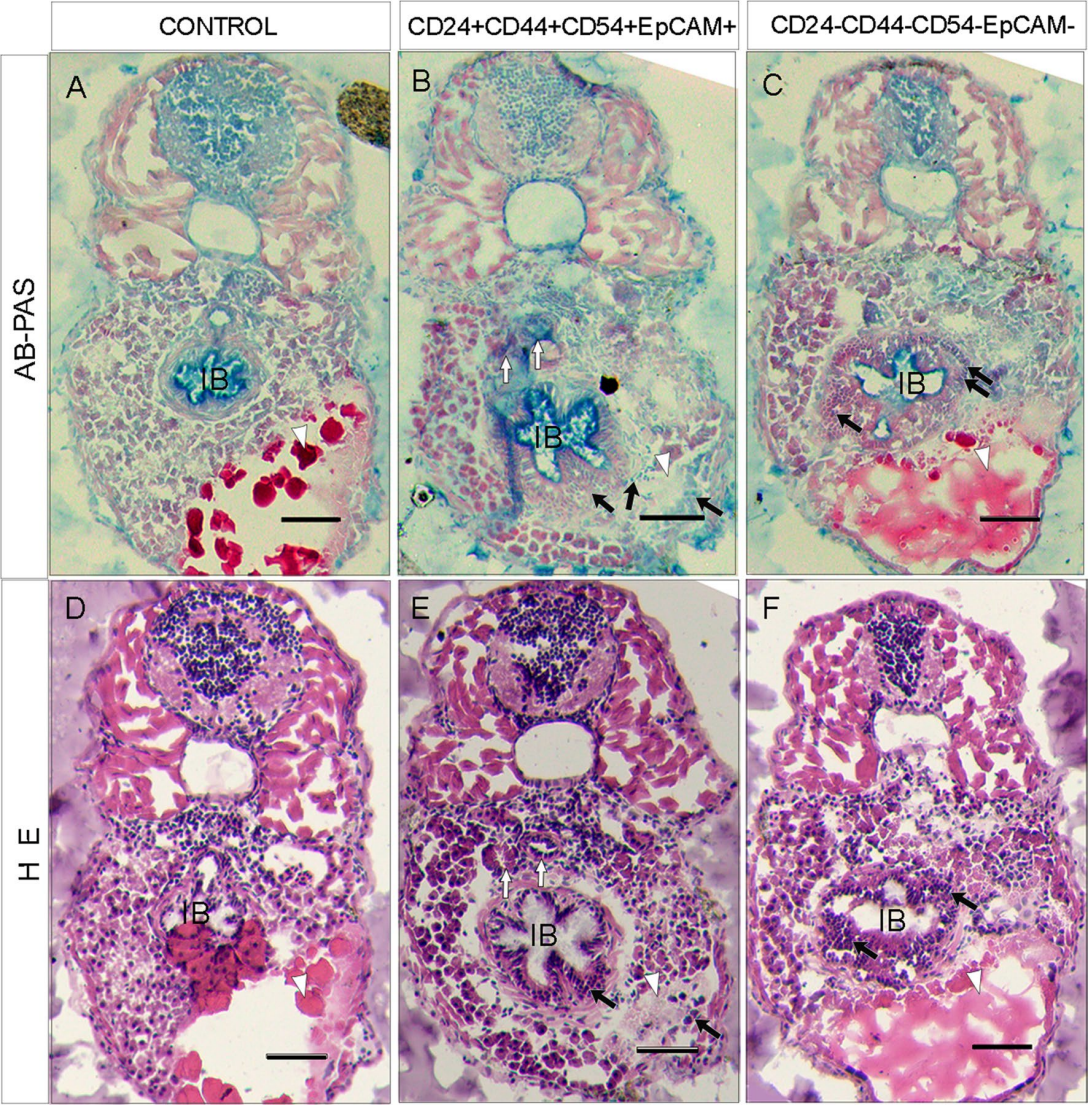
Mucin expression in cells derived from injected zebrafish. **A**–**F** Transversal sections near the level of injection site. **A**–**C** Alcian blue-PAS staining images of serial histological slices (AB-PAS). **A**, **D** Uninfected zebrafish. **B**, **E** Zebrafish transversal sections 4 dpi with GCEP cells. White arrows indicate misplaced cellular structure similar to blood vessels structures. Black arrows show tumor cell structures. **C**, **F** Transversal section of a zebrafish injected with GCnEP cells. The black arrows in **B**, **C**, **E**, **F** show tumor cell mass disrupting the intestinal bulb. Lipids and proteins from the vitelum (white arrowheads). All scale bars indicate 100 μM. Images were acquired with a ZEISS Axio Zoom V16 and processed in Zen 2.6 blue edition, Zeiss

The larvae injected with GCEPs showed massive changes in tissue structure. To characterize these changes, we performed AB-PAS staining (Fig. 5A–C) or H&E staining (Fig. 5D–F) of transverse sections of 4 dpi larvae.

Figure 5B, E shows tumor formation in the IB and vitelum (white arrowhead) in GCEP-injected larvae. Changes in structure can be observed in the IB and the muscle cells surrounding it compared to control larvae (Fig. 5A, D). In addition, the presence of misplaced cellular structures similar to blood vessels (white arrows in Fig. 5B, E), which were not observed in control larvae, could suggest the proangiogenic capacity of the GCEPs. GCnEPs (Fig. 5D, F) also had the ability to induce tumor growth in the IB and disruption of the muscle surrounding it, although they had a much lower metastatic capability.

PAS-AB staining shows the presence of mucins, a type of glycoprotein found throughout the gastrointestinal epithelia [30]. Changes in mucin production have been associated with cancer subtype [31, 32] and progression [33]. After injection into zebrafish, the tumors derived from GCEPs and GCnEPs had different mucins (black arrows, Fig. 5B, C). We found that the larvae injected with GCEPs exhibited mainly neutral mucins (PAS-stained mucins in magenta) and a few cells with acidic mucins (AB-stained mucins) (black arrows in Fig. 5B). Conversely, the larvae injected with GCnEPs showed areas with acidic mucin production and regions with production of a mixture of acidic and neutral mucins (two black arrows and a single black arrow, respectively, Fig. 5C). These findings are consistent with previous reports indicating heterogeneous phenotypes and expression of mucins during cancer progression in patients with GC [30–33].

Notably, larvae injected with GCEPs are almost devoid of vitelum (which contains a supply of protein and lipids to sustain metabolic functions and growth; white arrowheads in Fig. 5), in contrast with control or GCnEP-injected larvae. This suggests higher metabolic activity of the injected GCEPs.

## Discussion

In this study, we identified an extended phenotype of GCSCs derived from panendoscopy-extracted tissue samples from a cohort study of patients with GC. Eight CSC surface markers were evaluated in gastric tissue samples using multistaining protocols to find an extended GCSC phenotype that would allow for a better understanding of gastric carcinogenesis as well as more specific identification of GCSCs to design new and efficient approaches to treat GC patients. Our data demonstrate the presence of a subset of cells with a CD24+CD44+CD54+EpCAM+ phenotype in gastric adenocarcinomas from 127 patients. Although the mean age of the patients included in this study was 56.12 years (Table 1), our data come from a population limited to patients who attended the Instituto Nacional de Cancerología. Therefore, we cannot make any conclusions about the lower median age for GC diagnosis in Mexico compared to the average age of GC diagnosis published by the American Cancer Society, which is 68 years old.

It has been established that CSCs possess unique abilities, not only related to self-renewal and differentiation mechanisms but also related to cellular plasticity, which allows adaptation to new environments, which is crucial for the establishment of cells at distant sites [34]. Our data demonstrate a strong positive relationship between the percentage of GCEPs and metastasis in patients with GC, probably due to changes in the migration and invasion abilities of GCSCs through the acquisition of a transient EMT phenotype [35], in addition to stemness. At this time, we lack data to confirm whether the observed metastasis is hematogenous or transcellular metastasis. These results encouraged us to initiate a phase I diagnostic test study for GC detection through the assessment of circulating CD24+CD44+CD54+EpCAM+ cells, which is currently ongoing by our group.

We demonstrated that GCEPs have self-renewal capacity, tumor formation capacity, and differentiation potential in vitro, thereby producing cells with different surface markers derived from single cells with the CD24+CD44+CD54+EpCAM+ phenotype. This indicates that this subset of cells has a true GCSC phenotype. Moreover, GCEPs xenotransplanted into zebrafish showed higher tumor formation potential but also higher invasion and metastatic capacity compared to GCnEPs (Fig. 3A–D) and the formation of metastatic tumors in the distal portion of the intestine (Fig. 3C). In vivo, xenotransplanted GCEPs had a mesenchymal-like appearance, both outside and inside the blood vessels (Figs. 3B, 4C, E). The confocal images in Fig. 4 show the presence of CM-DiI-red- and yellow-stained cells, which are morphologically mesenchymal-like cells, both outside and inside the blood vessels. These observations support the hypothesis that the CD24+CD44+CD54+EpCAM+ phenotype in GCSCs is important for EMT and, consequently, for the migration, invasion, and high metastatic potential observed both in vitro and in vivo in zebrafish, as well as in patients with GC. Furthermore, since CM-DiI dye allows multigenerational monitoring of cells, it was notable that xenotransplanted CD24+CD44+CD54+EpCAM+ GCSC cells showed a range of fluorescence intensities at 6 dpi (Fig. 4C, D), suggesting that some cells are proliferating, resulting in lower concentrations of CM-Dil dye in those cells (faint yellow, arrowheads in Fig. 4C). These observations fit the hierarchical CSC model, which indicates that CSCs are primarily in quiescence [36] but that they can also proliferate and migrate to promote metastasis. Additionally, the presence of these GCSCs was strongly associated with the GC TNM clinical stage but was not related to overall survival in our patient sample. Consequently, in addition to the lack of evaluation of the tumorigenic capacity of cells with all surface marker combinations involving our four markers, CD24, CD44, CD54, and EpCAM, the limitations of this study are related to loss of contact with patients diagnosed with GC who were included in this protocol, since follow-up to determine patient survival can be complicated.

Furthermore, the evaluation of CD24+CD44+CD54+EpCAM+ GCSCs in patients with GC showed that the presence of these cells is significantly associated with metastasis in patients. This finding indicates that CD24+CD44+CD54+EpCAM+ cells could be an additional prognostic tool for the early detection of metastasis. This creates an opportunity to perform targeted therapy against these cells in GC patients.

As previously reported, the CD24 and CD44 cell surface proteins have been recognized as CSC markers in other cancers [23, 37]. The CD24+CD44+ subpopulation in GC cell lines has shown highly tumorigenic properties, self-regeneration, and multilineage differentiation in vitro [13, 14]. CD24 is frequently overexpressed in different types of cancer, such as ovarian, breast, prostate, bladder, and renal cancer, and is correlated with poor prognosis [11, 12, 23, 38]. CD44 is associated with other proteins that are used to monitor changes in the extracellular matrix and that specifically regulate important processes such as cell adhesion, proliferation, cell growth, survival, migration, angiogenesis, and in some cases, differentiation [39]. Our data show that CD24, CD44, and EpCAM were coexpressed with CD54 in all patient-derived tissues.

CD44 constitutes a cell surface adhesion molecule and the receptor for the hyaluronan glycan, which is expressed by a variety of cells, including those of the gastric epithelium, and may be implicated in gastric carcinogenesis. As the principal ligand of CD44, hyaluronan acts as an important player in the modulation of intracellular signaling pathways. Hyaluronan-mediated CD44 activation requires posttranslational modifications such as glycosylation of the extracellular domain and/or phosphorylation of specific serine residues in the cytoplasmic tail of the receptor. Interestingly, CD44 glycoforms containing sialyl-Tn precursor structures (STn) have been identified in the serum of GC patients [33]. One isoform of this receptor, CD44v6, has been associated with tumor progression and metastasis; a high percentage of CD44v6-positive cells indicates poor survival in several cancers, including GC [25].

The combination of the CD44+ and CD54+ markers has been used for the identification of CSCs in GC since both biomarkers are associated with metastasis, tumor recurrence, and mortality. Chen et al. demonstrated that a CD44+CD54+ cell subpopulation from samples from patients with GC has the ability to form tumorspheres and undergo self-renewal and shows greater tumorigenic potential than the CD44−CD54− subpopulation [7]. Furthermore, CD54 expression is enriched after chemotherapy and may contribute to chemoresistance. The authors also determined that CD54+ prostate cancer cells show CSC characteristics in vitro using sphere-forming and colony formation assays and in vivo using a patient-derived xenograft mouse model [40]. To the best of our knowledge, the role of CD24, CD44, CD54, and EpCAM in combination to identify CSCs in GC has not been evaluated within the same cell populations in cell lines and patient-derived tissues. We propose that this extended phenotype for the identification of GCSCs enables more precise isolation to elucidate the mechanisms that allow GCSCs to maintain their increased tumorigenic capacity, allowing the specific design of new approaches for oncoimmunotherapy and chemotherapy for the care of patients with GC. Targeting GCSCs may improve the antineoplastic response and prevent severe side effects in cancer patients. Furthermore, given that the data presented here could be relevant for making decisions regarding antineoplastic treatments to prevent metastasis, it will be interesting to open this study to international patients with GC to validate these findings. To reach a more solid conclusion and give more clinical value to the assessment of CD24+CD44+CD54+EpCAM+ GCSCs for prognosis of this disease, it will be necessary to obtain complete patient clinical data for CD24+CD44+CD54+EpCAM+ GCSCs from a larger number of patients to determine their relationship with overall or disease-free survival.

We used zebrafish to study cells with CSC characteristics. One of the major advantages of this model is avoiding the use of immunosuppressants [41]. In this study, we injected 50 or 200 GCEPs or GCnEPs into zebrafish embryos at 48 hpf. Notably, after 1 dpi, we observed that only the GCSCs with the CD24+CD44+CD54+EpCAM+ phenotype had migrated from the yolk sac to the tail of the larvae, showing strong tumorigenic potential (Fig. 3A). We also found metastatic tumors in the distal portion of the intestine at 6 dpi (Fig. 3C). The cells were able to survive, extravasate, and colonize distant regions at only 1 dpi, in contrast with the CD24−CD44−CD54−EpCAM− cells (Fig. 3A panels I and II), which required 6 dpi to exhibit slight migration. We also observed the capacity of these cells to acquire a transient EMT phenotype, in which the cells lose their polarity and cell-to-cell contacts, showing a mesenchymal phenotype (Fig. 4E) with invasive characteristics. CD24+CD44+CD54+EpCAM+ GCSCs infiltrated the blood vessels and entered the circulation, showing enhanced migration and invasion capabilities [42] (Fig. 4B–D). Our in vitro assays show that CD24+CD44+CD54+EpCAM+ GCSCs exhibit the ability to differentiate and generate different subsets of cells with distinct phenotypes, such as the CD24−CD44−CD54-EpCAM− phenotype, similar to the results observed in the in vivo model, where the CD24+CD44+CD54+EpCAM+ GCSCs exhibited differentiation potential, generating specialized cells with the ability to secrete mucins (Fig. 5), a characteristic described for some GC types that indicates deeper invasion, greater size and higher metastatic potential.

## Conclusion

Overall, we have demonstrated in patient GC tissue biopsies and in cells derived from the AGS cell line the presence of a subset of cells with an extended CSC phenotype, CD24+CD44+CD54+EpCAM+; these cells possess self-renewal capacity, cell differentiation potential, stemness, and maximal tumorigenicity. These phenotypic and functional stemness characteristics make CD24+CD44+CD54+EpCAM+ true GCSCs. This extended phonotype could improve the design of new, targeted therapies to promote the elimination of these highly tumorigenic cells. Additional data presented in this work suggest that an increased percentage of CD24+CD44+CD54+EpCAM+ GCSCs is closely related to metastasis in patients with GC (Table 2). Adding this assessment to alternative tools used to select the best treatment for this neoplasm could be useful in determining the possibility of metastasis and may have prognostic value. Therefore, the evaluation of CD24+CD44+CD54+EpCAM+ GCSCs may support new therapeutic approaches in precision medicine, resulting in improved healthcare for GC patients. Finally, to reach a more solid conclusion and give more clinical value to the assessment of CD24+CD44+CD54+EpCAM+ GCSCs for the prognosis of this disease, it will be necessary to obtain complete patient clinical data on CD24+CD44+CD54+EpCAM+ GCSCs from a larger number of patients to determine their relationship with overall or disease-free survival.

**Table 2:** Main metastasis sites in patients with gastric cancer

Metastasis site	*n* = 127 (%)
Without evidence	75 (59.1%)
Liver	19 (15%)
Lung	11 (8.7%)
Retroperitoneum	4 (3.1%)
Ovary	4 (3.1%)
Colon	3 (2.4%)
Adrenal gland	3 (2.4) %)
Bone	2 (1.6%)
Mediastinum	2 (1.6%)
Distant abdominal nodes	2 (1.6%)
Pancreas	1 (0.8%)
Spleen	1 (0.8%)

## Supplementary Information

**Additional file 1: Figure S1.** Percentage of CD24+CD44+CD54+EpCAM+ cells on different days of culture in AGS cells. Cells from the AGS cell line were cultured in non-adherent conditions, without supplemented media, and then harvested for flow cytometry analysis for different cancer stem cell markers on day 0, 1, 3, 5 and 7 of culture. We observed an increase in the CD24+CD44+CD54+EpCAM+ on day 3 compared with other days. Ten thousand cells were acquired by the NXT Attune cytometer. Error bars indicate the ± SD of three independent assays. *P ≤ 0.05.

**Additional file 2:** **Figure S2.** Presence of CD73, CD90, CD184 and STRO-1 on cells from gastric cancer patients. We analyze the presence of the Cancer Stem Cell markers CD73, CD90, CD184 and STRO-1 in the population CD24+CD44+, however we did not identify co-expression of these markers in the subpopulation CD24+CD44+. The data represents the strategy of analysis of one patient. Error bars indicate the ± SD of three independent assays. *P ≤ 0.05.

**Additional file 3:** **Figure S3.** GCEP cells are also represented in tumorspheres derived from different cell lines. Cells from the KATO-III and NCI-N87 cell lines were cultured in non-adherent condition without supplemented media, and then harvested for flow cytometry analysis using the CD24, CD44, CD54 and EpCAM on days 0, 1, 3, 5 and 7 of culture. As same as with the AGS cell line, we observed and increase on the population CD24+CD44+CD54+EpCAM+ on day 3 compared with the other days. Error bars indicate the ± SD of three independent assays. *P ≤ 0.05.

**Additional file 4:** **Figure S4.** Stemness markers are increased in CD24+CD44+CD54+EpCAM+ cells. Day 3 tumorspheres were dispersed and single cell suspensions were stained for multiparametric flow cytometry. Cells were stained with CD24, CD44, CD54, and EpCAM antibodies, then the analysis of the expression of the stemness markers was performed in the CD24+CD44+CD54+EpCAM+ subpopulation. (A) OCT4 expression in CD24+CD44+CD54+EpCAM+ . We observed 96% of cSCRT-D-22–00745.ells were positive for OCT4, compared to 24% in the CD24−CD44−CD54−EpCAM− subpopulation. (B) SOX2 expression in CD24+CD44+CD54+EpCAM+ cells. We observed 55% of cells were positive, but there was only 17% SOX2 positive cells in the negative subpopulation. (C) NANOG expression in CD24+CD44+CD54+EpCAM+ cells. We found 45% of the cells expressing NANOG, while only 15% of the CD24−CD44−CD54−EpCAM− cells express NANOG. In all the three transcription factors, OCT4, SOX2 and NANOG, we observed an increased expression of these in the subpopulation CD24+CD44+CD54+EpCAM+ compared with the negative population. *P ≤ 0.05.

**Additional file 5:** **Figure S5.** Zebrafish with GCEP cells have lower survival rate and higher migration compared to zebrafish with GCnEP cells. (**A**) 200 GCEP or GCnEP sorted cells stained with CM-DiI dye were injected into Zebrafish embryos of 48 hpf. After 1 dpi GCEP cells migrated from the yolk to the tail. (**B**) Overall survival of zebrafish embryos injected with 200 GCEP cells compared to embryos injected with GCnEP cells. Control embryos were injected with PBS.

## Data Availability

The datasets of the present study are available from the corresponding author upon reasonable request.

## Abbreviations

ANOVA: Analysis of variance
APC: Allophycocyanin
ATCC: American Type Culture Collection
bFGF: Basic fibroblast growth factor
BD: Becton Dickinson
CICUAL: Comité Institucional para el Cuidado y Uso de Animales de Laboratorio
DPI: Days post-injection
EGF: Epidermal growth factor
EGFP: Enhanced green fluorescent protein
EpCAM: Epithelial cellular adhesion molecule
EMT: Epithelial–mesenchymal transition
FACS: Fluorescence-activated cell sorter
FBS: Fetal bovine serum
FITC: Fluorescein isothiocyanate
GC: Gastric carcinoma
CSCs: Cancer stem cells
GCEP: Gastric cancer cells with extended phenotype
GCnEP: Gastric cancer cells with negative extended phenotype
GCSCs: Gastric cancer stem cells
Hpf: Hours post-fertilization
Hpi: Hours post-injection
H&E: Hematoxylin and eosin
IB: Intestinal bulb
IFC: Instituto de Fisiología Celular
IMPI: Instituto Mexicano de la Propiedad
IIB: Instituto de Investigaciones Biomédicas
PAS-AB: Periodic acid-Schiff–Alcian blue
PE: Phycoerythrin
PE-Cy5: Phycoerythrin-cyanine 5
PE-Cy7: Phycoerythrin-cyanine 7
PBS: Phosphate-buffered saline
PTU: Phenylthiourea
SEM: Standard error of the mean
SOX2: Sex-determining region Y box 2
STn: Sialyl-Tn precursor structures
UNAM: Universidad Nacional Autónoma de México
LabNalCit: Laboratorio Nacional de Citometría
TF: Transcription Factors0020
Tg: Transgenic
OCT4: Octamer-binding transcription factor 4
WHO: World Health Organization
