# Supplementary figures and images for: CD24+CD44+CD54+EpCAM+ gastric cancer stem cells predict tumor progression and metastasis: clinical and experimental evidence
Source: Stem Cell Res Ther. 2023 Feb 3;14:16. doi: 10.1186/s13287-023-03241-7 (PMC9898964; doi:10.1186/s13287-023-03241-7)

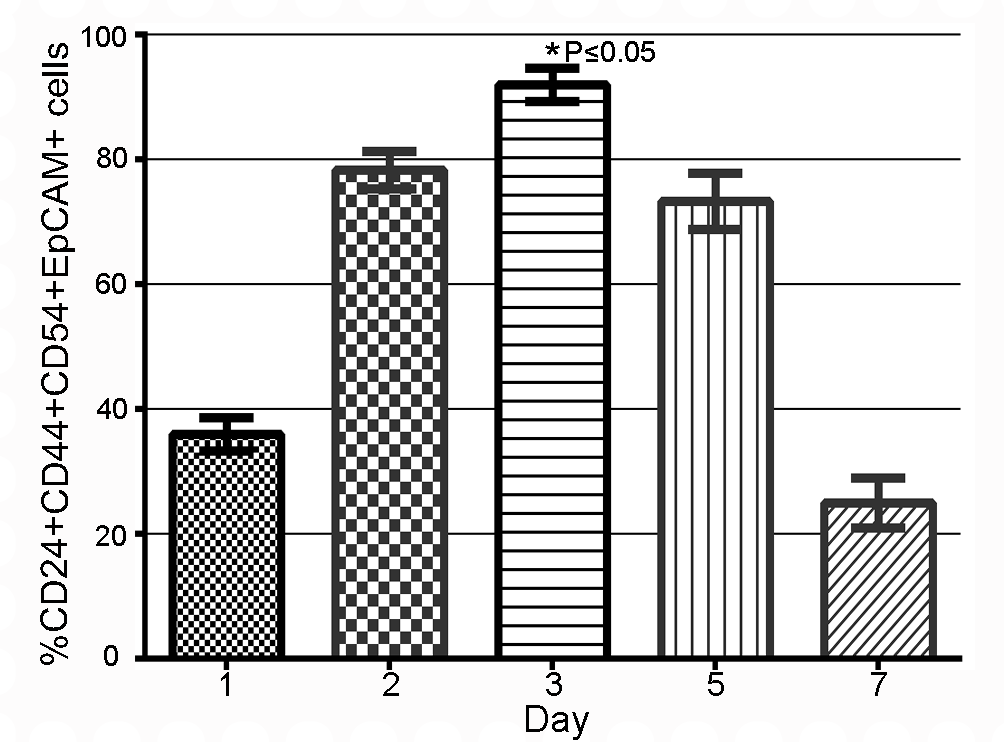

Supplement: Supplementary file 1 — Additional file 1: Figure S1. Percentage of CD24+CD44+CD54+EpCAM+ cells on different days of culture in AGS cells. Cells from the AGS cell line were cultured in non-adherent conditions, without supplemented media, and then harvested for flow cytometry analysis for different cancer stem cell markers on day 0, 1, 3, 5 and 7 of culture. We observed an increase in the CD24+CD44+CD54+EpCAM+ on day 3 compared with other days. Ten thousand cells were acquired by the NXT Attune cytometer. Error bars indicate the ± SD of three independent assays. *P ≤ 0.05. [file 13287_2023_3241_MOESM1_ESM.tiff]

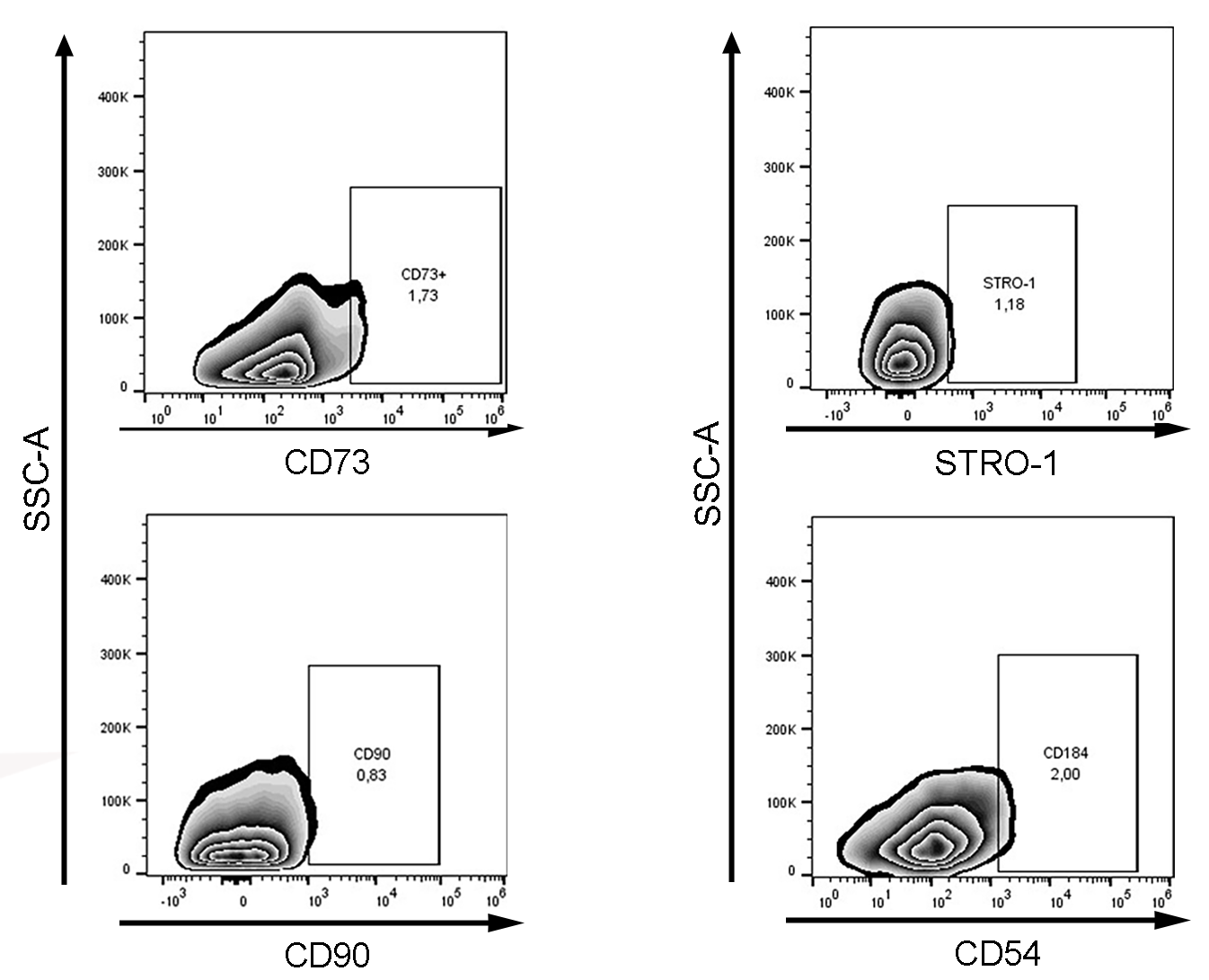

Supplement: Supplementary file 2 — Additional file 2: Figure S2. Presence of CD73, CD90, CD184 and STRO-1 on cells from gastric cancer patients. We analyze the presence of the Cancer Stem Cell markers CD73, CD90, CD184 and STRO-1 in the population CD24+CD44+, however we did not identify co-expression of these markers in the subpopulation CD24+CD44+. The data represents the strategy of analysis of one patient. Error bars indicate the ± SD of three independent assays. *P ≤ 0.05. [file 13287_2023_3241_MOESM2_ESM.tiff]

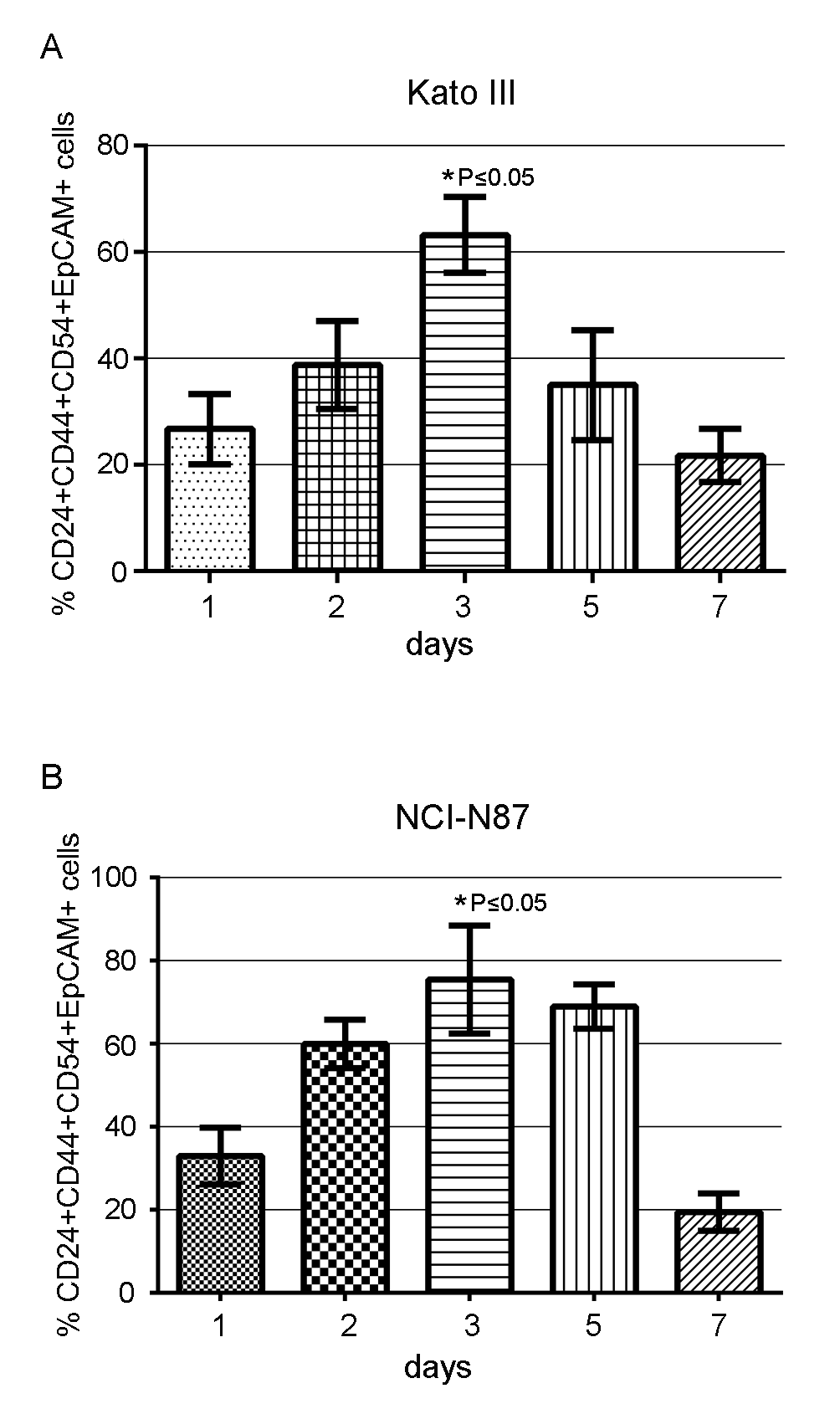

Supplement: Supplementary file 3 — Additional file 3: Figure S3. GCEP cells are also represented in tumorspheres derived from different cell lines. Cells from the KATO-III and NCI-N87 cell lines were cultured in non-adherent condition without supplemented media, and then harvested for flow cytometry analysis using the CD24, CD44, CD54 and EpCAM on days 0, 1, 3, 5 and 7 of culture. As same as with the AGS cell line, we observed and increase on the population CD24+CD44+CD54+EpCAM+ on day 3 compared with the other days. Error bars indicate the ± SD of three independent assays. *P ≤ 0.05. [file 13287_2023_3241_MOESM3_ESM.tiff]

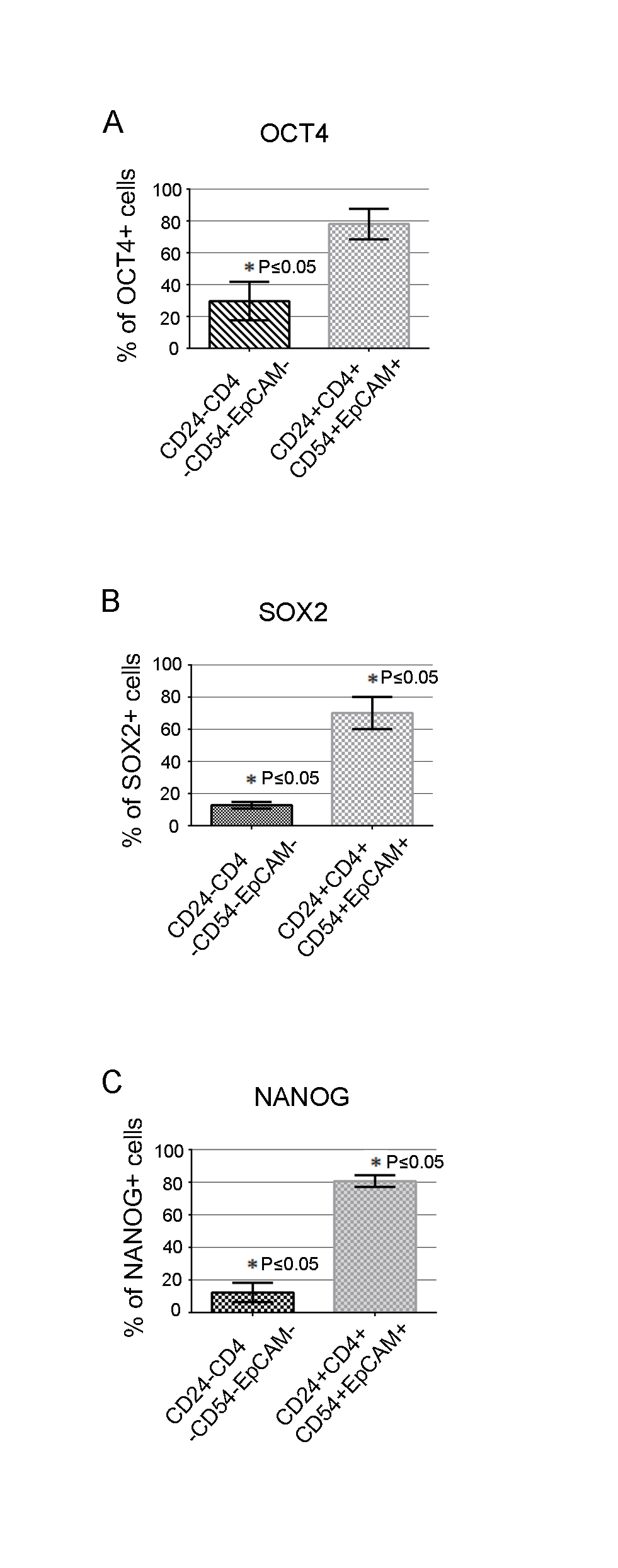

Supplement: Supplementary file 4 — Additional file 4: Figure S4. Stemness markers are increased in CD24+CD44+CD54+EpCAM+ cells. Day 3 tumorspheres were dispersed and single cell suspensions were stained for multiparametric flow cytometry. Cells were stained with CD24, CD44, CD54, and EpCAM antibodies, then the analysis of the expression of the stemness markers was performed in the CD24+CD44+CD54+EpCAM+ subpopulation. (A) OCT4 expression in CD24+CD44+CD54+EpCAM+ . We observed 96% of cSCRT-D-22–00745.ells were positive for OCT4, compared to 24% in the CD24−CD44−CD54−EpCAM− subpopulation. (B) SOX2 expression in CD24+CD44+CD54+EpCAM+ cells. We observed 55% of cells were positive, but there was only 17% SOX2 positive cells in the negative subpopulation. (C) NANOG expression in CD24+CD44+CD54+EpCAM+ cells. We found 45% of the cells expressing NANOG, while only 15% of the CD24−CD44−CD54−EpCAM− cells express NANOG. In all the three transcription factors, OCT4, SOX2 and NANOG, we observed an increased expression of these in the subpopulation CD24+CD44+CD54+EpCAM+ compared with the negative population. *P ≤ 0.05. [file 13287_2023_3241_MOESM4_ESM.tiff]

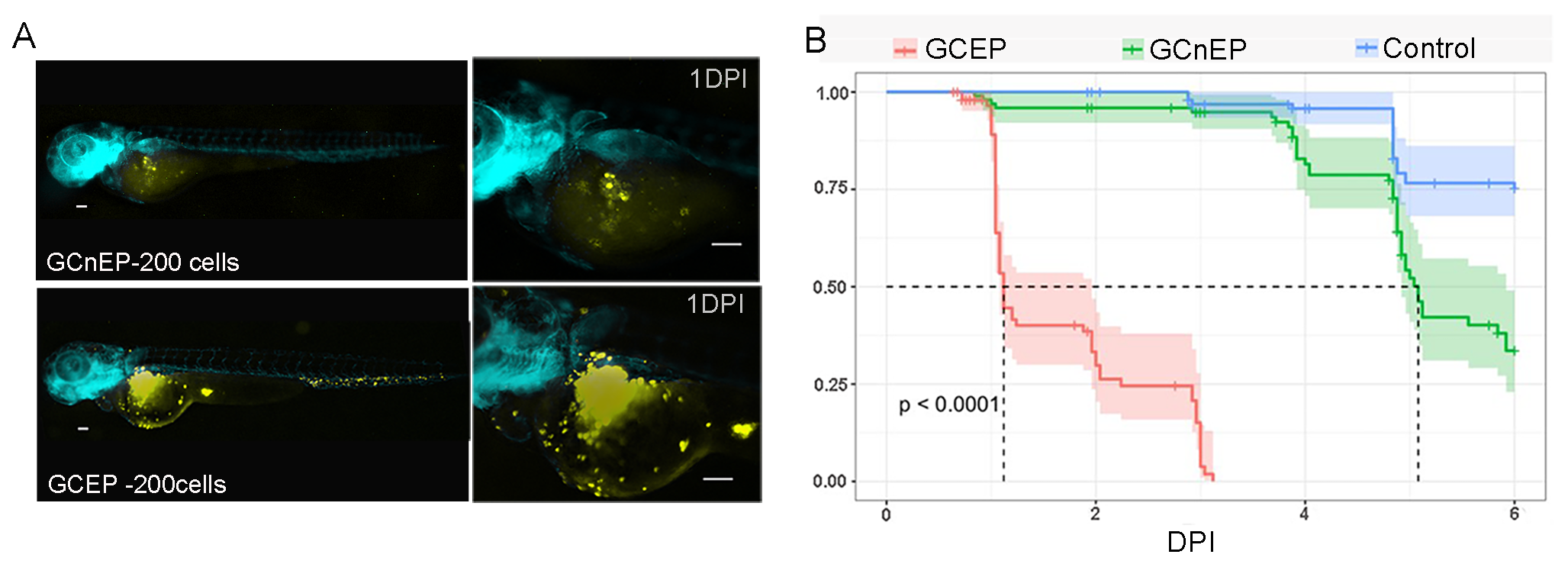

Supplement: Supplementary file 5 — Additional file 5: Figure S5. Zebrafish with GCEP cells have lower survival rate and higher migration compared to zebrafish with GCnEP cells. (A) 200 GCEP or GCnEP sorted cells stained with CM-DiI dye were injected into Zebrafish embryos of 48 hpf. After 1 dpi GCEP cells migrated from the yolk to the tail. (B) Overall survival of zebrafish embryos injected with 200 GCEP cells compared to embryos injected with GCnEP cells. Control embryos were injected with PBS. [file 13287_2023_3241_MOESM5_ESM.tiff]
